# Unlocking the training process: how different training tasks shape the physiology and biomechanics of professional futsal players

**DOI:** 10.3389/fspor.2025.1549026

**Published:** 2025-09-11

**Authors:** Carlos Serrano, António Ferraz, João Nuno Ribeiro, Enrique Ibañez, Bruno Travassos

**Affiliations:** ^1^ Department of Sports Sciences, Faculty of Medicine, Health and Sports, Universidad Europea de Madrid, Madrid, Spain; ^2^ Department of Sport Sciences, University of Beira Interior, Covilhã, Portugal; ^3^ CIFD, Sports Research and Training Center, Jean Piaget University of Angola, Luanda, Angola; ^4^ SPRINT Sport Physical Activity and Health Research & Innovation Center, Polytechnic Institute of Guarda, School of Education, Communication and Sports, Guarda, Portugal; ^5^ University of Las Palmas de Gran Canaria, Las Palmas de Gran Canaria, Spain; ^6^ Portugal Football School, Portuguese Football Federation, Oeiras, Portugal

**Keywords:** team sport, performance, workload, data visualization, LPS

## Abstract

**Introduction:**

Despite the characterization of the physical and technical tactical demands of futsal SSCGs, there remains a need to better understand the physiological and biomechanical loads of each training task to determine its workload during training sessions further. The present study aims to analyze and classify the physiological and biomechanical demands of different futsal training tasks to enhance the understanding of these demands and their implications for elite futsal players’ performance.

**Methods:**

Conducted over three seasons with a professional futsal team, this study systematically categorized training exercises into six task types: introductory, analytical, mid-court, ¾ court, full-court, and superiority/inferiority tasks. The WIMU PRO™ device was used to monitor physiological and biomechanical metrics and to assess how variations in task type influence them.

**Results:**

Superiority/inferiority tasks, followed by full-court tasks, showed significantly higher physiological and biomechanical loads compared with introductory, analytical, and mid-court tasks. These differences (*p* ≤ 0.05) were associated with higher values of average heart rate, total distance covered, and high-speed running per minute for the physiological load, as well as a greater number of accelerations, decelerations, and player load per minute for the biomechanical load.

**Discussion:**

A visual four-quadrant effort assessment provided insights into the contribution of each task category through its specific load distribution, revealing significant variations associated with player numbers, field dimensions, and task objectives. These findings suggest that specific task configurations can be strategically used to optimize training outcomes by aligning physical demands with coaching objectives.

## Introduction

Futsal is a team sport characterized by high dynamism and continuous interchange of players’ positions during the game. It is played at a high pace, with intermittent actions (1) that include repeated high-speed efforts, such as sprints, as well as high-intensity movements including accelerations, decelerations, changes of direction, and one-on-one situations (2). However, these physical demands result from specific individual and collective offensive and defensive tactical skills that require players to continuously adjust their positions and movements in relation to teammates and opponents (3).

Accordingly, training programs must integrate both tactical and physical components to optimize player performance regarding the high-intensity individual actions required in competition (4). Several studies have highlighted the importance of designing specific training tasks that replicate real-game scenarios to maximize training effectiveness (5, 6).

Therefore, to better understand the tactical and physical effects of each training task on player development, it is crucial to evaluate the workload generated (7). Properly distributing this workload across training sessions and the microcycle helps optimize players’ preparation for competition and recovery.

In team sports, particularly futsal, small-sided and conditioned games (SSCG) are widely used as a training method to develop players’ technical, tactical, and physical capacities, while promoting the transfer of these skills to competitive contexts (3). For example, research has shown that altering the number of players while maintaining a consistent playing space (40 m × 20 m) results in different physical demands, with distinct impacts on kinematic and mechanical high-intensity efforts (8). The use of large relative surfaces per player (30 m × 20 m and 40 m × 20 m) and a reduced number of players (two and three players) tends to increase the external and internal loading of futsal players compared with match demands (9). For instance, that study found that training tasks with larger relative areas increased the total distance covered. To promote high-intensity distances as well as high-intensity accelerations and decelerations, it is advisable to use medium-to-large relative areas (30 m × 20 m−40 m × 20 m) and structures such as GK + 2 vs 2 + GK or GK + 3 vs 3 + GK.

Recently, a study in rink hockey applied a four-quadrant effort typology to evaluate different task categories that training sessions had distinct biomechanical and physiological demands across tasks and were inadequate when compared with the competition (10). However, to the best of our knowledge, no similar study has been conducted in futsal. Despite the characterization of the physical and technical tactical demands of SSCGs, there is a need to really understand the physiological and biomechanical loads of each training task to clarify their contribution to overall workload.

Therefore, this study aims to analyze the workload typology of professional futsal training tasks to understand their impact on the physiological and biomechanical demands of players. Additionally, the study seeks to classify these training tasks using a visual quadrant-based approach to better clarify the types of efforts represented by each task category. It was hypothesized that different task profiles would emerge according to the training objectives, number of players involved, and pitch dimensions.

## Material and methods

### Participants

A total of 14 professional futsal players (age 29.07 ± 4.23 years; height 1.78 ± 0.06 m; weight 73.28 ± 4.30 kg) participated in this study. Goalkeepers were excluded from the analysis due to their distinct training categories and match requirements. Data collection adhered to international ethical standards for research involving humans, as outlined in the Declaration of Helsinki (11). Data were collected inside the club's performance analysis protocol, and all the players were identified with a code to ensure anonymity. The club provided written informed consent to use the data for research purposes. The study was approved and followed the procedures approved by the ethical committee of Universidade da Beira Interior CE-UBI-Pj-2020-043.

### Study design

An observational study was conducted with a team competing in the First Division of the Spanish futsal league during three consecutive seasons (2020–2021, 2021–2022, and 2022–2023). The tasks performed during the training sessions were classified and categorized based on previous literature (7) and using specific criteria (Table 1). These categories included introductory technique-activation exercises; analytical situations; mid-court exercises (20 m × 20 m); ¾ court exercises (28 m × 20 m); full-court exercises (40 m × 20 m); and superiorities/inferiorities. During the data collection, no instructions were given to the technical staff regarding exercise selection or in-match coaching decisions.

**Table 1:** Task description for each analyzed category.

Training task	Task description
Introductory technique-activation exercises (20 × 20; 28 × 20, 40 × 20)	Upper- and lower-body activation Strength and mobility exercises Movements activation displacements without ball Manipulating the ball, associating passes with/without opposition Introductory games without or with ball Finalization to goalkeeper warm-up individual
Analytical situations (20 × 20; 40 × 20)	Specific tactical movements with/without opponents with passes, and finalization (i.e., shooting)
Mid-court (20 × 20 m)	Game with/without goalkeeper in 2 vs. 2 and 3 vs. 3 formats Game with/without goalkeeper in 2 vs. 2 and 3 vs. 3 formats with outfield player jocker Exercises involving outfield goalkeeper attacking and defensive actions
¾ of the court (28 × 20 m)	Game with goalkeeper in 2 vs. 2, 3 vs. 3, and 4 vs. 4 formats with/without players jocker
Full-court (40 × 20 m)	Game with goalkeeper (4 vs. 4 and 5 vs. 5 formats)
Superiorities/inferiorities (40 × 20)	Situations with numerical superiorities/inferiorities (2 × 1, 3 × 1, and 3 × 2)

### Measures

Players’ activity during training sessions was monitored using the WIMU PRO™ device (Realtrack Systems SL, Almeria, Spain), which employs inertial measurement units (IMUs), an ultra-wideband (UWB) local positioning system (LPS) technology, and a heart rate sensor (Garmin Ltd., Olathe, KS, United States). Additionally, all training sessions were recorded with a video camera (Sony, Tokyo, Japan). All recorded data were downloaded and subsequently processed using the corresponding software (SPRO™, Realtrack Systems SL, Version 989), synchronized with the video footage to adjust for active times and player participation in each task.

### Physiological and biomechanical variables

Internal and external data (Table 2) were collected and categorized into physiological and biomechanical variables (10). Data were expressed as relative values per minute (min^−1^), and only the periods in which players actively participated in the exercises were considered.

**Table 2:** Physiological and biomechanical variables recorded in this study.

Type	Variable	Unit	Description
Time practice	Exercise/match duration	TMOP (min)	Total time of practice in minutes
Physiological	Heart rate	HR_AVG_ (bpm)	Average heart rate (bpm)
	Total distance covered	TD_min−1_ (m)	Total distance running per minute in meters
	High-speed running	HSR_min−1_ (m)	Total distance running between 15 and 30 km/h per minute
Biomechanical	High-intensity accelerations	ACC_min−1_ (3–10 m/s^2^) (m)	Total distance covered in positive speed changes per minute
	High-Intensity Decelerations	DEC_min−1_ (−10 to −3 m/s^2^) (m)	Total distance covered in negative speed changes per minute
	Player load	PL_min−1_ (a.u.)	Accumulated accelerometer load in the three axes of movement per minute
	Metabolic power	MP (W/kg/min)	Metabolic power per kilogram of body weight per minute

To create a visual interpretation of physiological and biomechanical demands, a cluster analysis was performed to group observations based on the similarity of selected variables. Cluster membership was then assigned, and each observation was classified accordingly. Each cluster's mean physiological and biomechanical values were then calculated, and the intersection of these means was used to determine the cluster's position within a four-quadrant visualization (10).

### Statistical analysis

*A priori* power analysis was conducted using G*Power (Version 3.1.9.2) to determine the necessary sample size, considering the following input parameters: effect size *d* = 0.8, *α* = 0.05, and statistical power = 0.9. The minimum required sample size was 8, which was achieved in the present study. A linear mixed model with random intercepts was used to compare differences in external [TD_min−1_ (m); HSR_min−1_ (m); ACC_min−1_ (m); DEC_min−1_ (m); PL_min−1_ (a.u.)] and internal [(HR_AVG_) and metabolic power (MP) (W/kg/min)] load across training tasks categories while accounting for repeated measures at the individual level. Training task categories were treated as categorical fixed effects, external and internal load variables were included as dependent variables, and subjects were included as random effects. *Post hoc* pairwise comparisons with Bonferroni correction were conducted to identify specific external and internal load differences between task categories. The residuals’ distribution was assessed after fitting a linear mixed model to verify normality deviation (12). Cohen's *d* ES and 95% confidence intervals (CI) were calculated and interpreted as <0.2 trivial; 0.20–0.59 small; 0.60–1.19 moderate; 1.2–1.99 large; and ≥2.0 very large (13). The significance level was set at *p* < 0.05.

In line with the study's goal, a two-step cluster analysis was conducted to classify training and match demands based on physiological and biomechanical stress, using log-likelihood as the distance measure and Schwartz's Bayesian criterion (14). To determine each exercise's physiological and biomechanical load, an average value was calculated for each task category based on the numerical score of the cluster ranging from 1 to 2 for physiological (e.g., 1, low physiological load; 2, high physiological load) and from 1 to 2 for biomechanical (e.g., 1, low biomechanical load; 2, high biomechanical load). The linear mixed model analysis was carried out using the statistical program Jamovi (Version 1.8, 2021), while the two-step cluster analysis was carried out using IBM SPSS Statistics for Windows (Version 28.0, IBM Corp., Armonk, NY, USA). A quadrant plot was used to visually illustrate the classification of each training task category.

## Results

We comprehensively compared the various exercise categories by analyzing multiple physiological and biomechanical variables, as shown in Table 3. For physiological load, introductory exercises revealed significantly lower (*p* ≤ 0.05) values for HR_AVG_, TD_min−1_, and HSR_min−1_ than all other categories. Additionally, ¾ court exercises showed the highest HR_AVG_ values. Full-court and superiorities/inferiorities exercises presented significantly higher values of TD_min−1_ when compared with introductory tasks. Superiorities/inferiorities and full-court exercises showed the highest significant (*p* ≤ 0.05) values of HSR_min−1_ when compared with the others.

**Table 3:** Linear mixed model and description of the physiological and biomechanical metrics by task categories.

Variables	Exercises categories												Total, *n* = 1,979
	Introductory technique-activation, *n* = 167 (95% CI)	ES	Analytical situations, *n* = 213 (95% CI)	ES	Mid-court, *n* = 362 (95% CI)	ES	¾ of the court, *n* = 682 (95% CI)	ES	Full-court, *n* = 340 (95% CI)	ES	Superiorities/inferiorities, *n* = 215 (95% CI)	ES	
TMOP (min)	10.84 ± 3.61 (10.29–11.40)		6.89 ± 1.67 (6.66–7.11)		7.06 ± 9.30 (6.10–8.02)		4.24 ± 1.95 (4.09–4.38)		3.87 ± 2.16 (3.64–4.10)		2.50 ± 0.54 (2.42–2.60)		5.35 ± 4.93 (5.13–5.56)
Physiological													
HR_AVG_ (bpm)	123.20 ± 23.37 (119.65–126.76)^a^^,^^b^^,^^c^^,^^d^^,^^e^	0.76 0.87 1.06 0.91 0.86	153.850 ± 17.84 (151.44–156.26)		152.24 ± 18.05 (150.38–154.11)		155.28 ± 18.27 (153.90–156.65)		153.13 ± 22.88 (150.69–155.57)		153.28 ± 17.70 (150.90–155.66)		151.23 ± 21.25 (150.34–152.21)
TDS_min−1_ (m)	63.18 ± 23.62 (59.57–66.79)^a^^,^^b^^,^^c^^,^^d^^,^^e^	0.45 0.56 0.64 0.95 0.93	85.21 ± 10.77 (83.75–86.66)^d^^,^^e^	0.51 0.49	87.61 ± 22.44 (85.29–89.93)^d^^,^^e^	0.50 0.51	89.31 ± 19.53 (87.84–90.78)^d^^,^^e^	0.53 0.52	105.62 ± 30.39 (102.37–108.85)		109.40 ± 14.99 (107.39–111.42)		91.34 ± 24.71 (90.25–92.23)
HSR_min−1_ (m)	3.73 ± 4.78 (3.00–4.46)^b^^,^^c^^,^^d^^,^^e^	0.24 1.15 0.85 1.21	3.77 ± 3.50 (3.30–4.25)^b^^,^^c^^,^^d^^,^^e^	0.23 0.42 0.89 1.27	8.05 ± 6.71 (7.36–8.75)^c^^,^^d^^,^^e^	0.19 0.76 1.19	10.82 ± 6.24 (10.35–11.29)^d^^,^^e^	0.68 1.15	20.63 ± 15.23 (19.00–22.25)^e^	0.51	29.75 ± 15.16 (27.71–31.79)		12.70 ± 12.35 (12.16–13.24)
Biomechanical													
ACC_min−1_ (m)	2.22 ± 2.90 (1.77–2.66)^a^^,^^b^^,^^c^^,^^d^^,^^e^	0.43 0.41 0.46 0.27 0.43	6.92 ± 5.25 (6.21–7.63)^d^^,^^e^	0.22 0.18	6.54 ± 5.95 (5.93–7.16)^d^^,^^e^	0.17 0.10	7.30 ± 4.22 (6.97–7.61)^d^^,^^e^	0.20 0.12	7.66 ± 6.04 (7.01–8.30)^e^	0.22	12.65 ± 7.72 (11.61–13.69)		7.33 ± 5.86 (7.07–7.59)
DEC_min−1_ (m)	2.15 ± 2.84 (1.71–2.58)^a^^,^^b^^,^^c^^,^^d^^,^^e^	0.48 0.42 0.44 0.27 0.32	6.67 ± 4.73 (6.03–7.30)^c^^,^^d^^,^^e^	0.15 0.28 0.18	6.27 ± 5.55 (5.69–6.84)^d^^,^^e^	0.18 0.07	6.16 ± 3.43 (5.90–6.42)^d^^,^^e^	0.19 0.09	6.94 ± 5.23 (6.38–7.50)		9.60 ± 6.23 (8.76–10.45)		6.40 ± 4.94 (6.19–6.62)
PL_min−1_ (a.u.)	1.08 ± 0.40 (1.02–1.14)^a^^,^^b^^,^^c^^,^^d^^,^^e^	0.53 0.70 0.68 0.87 0.80	1.64 ± 0.34 (1.60–1.69)^d^^,^^e^	0.30 0.27	1.71 ± 0.58 (1.65–1.77)^d^^,^^e^	0.22 0.20	1.64 ± 0.43 (1.61–1.68)^d^^,^^e^	0.35 0.31	1.87 ± 0.60 (1.81–1.94)		1.93 ± 0.34 (1.88–1.97)		1.68 ± 0.52 (1.66–1.70)
Metabolic power (W/kg/min)	2,298.79 ± 0.40 (1,771.65–2,825.92)^b^^,^^c^^,^^d^^,^^e^	0.15 0.14 0.19 0.24	1,751.14 ± 0.40 (1,664.40–1,837.88)^d^^,^^e^	0.13 0.19	1,516.42 ± 0.40 (1,340.12–1,692.72)^e^	0.13	1,101.83 ± 0.40 (1,047.70–1,155.96)^e^	0.16	1,164.05 ± 0.40 (1,102.80–1,225.30)		817.30± (792.38–842.21)		279.99 ± 221.30 (1,266.48–1,390.19)

Data presented as mean ± standard deviation and IC.

ES: Cohen's *d* effect size, reported only for significant differences and not reported for repeated significant differences. Repeated statistical differences between groups were not presented.

Time of practice [TMOP (min)]; average heart rate (HR_AVG_;); high-speed running per minute [HSR_min−1_(m)]; total distance running per minute [TD_min−1_ (m)]; distance covered in accelerations +3 m per minute [ACC_min−1_ (m)]; distance covered in deceleration +3 m per minute [DEC_min−1_ (m)]; player load per minute [PL_min−1_ (a.u.)]; power metabolic load per minute (W/kg/min).

^a^ Significantly different than ANLT (*p* ≤ 0.05).

^b^ Significantly different than MDE (*p* ≤ 0.05).

^c^ Significantly different than ¾ CE (*p* ≤ 0.05).

^d^ Significantly different than FCE (*p* ≤ 0.05).

^e^ Significantly different than SIE (*p* ≤ 0.05).

Regarding biomechanical load, a similar tendency within exercise categories was observed. Despite introductory exercises demonstrating higher MP values (*p* ≤ 0.05) than all categories, significantly lower values of ACC_min−1_, DEC_min−1_, and PL_min−1_ were observed in this category when compared with the others. Moreover, analytical, mid-court, and ¾ court tasks were characterized by displaying lower (*p* ≤ 0.05) values of ACC_min−1_, DEC_min−1_, and PL_min−1_ when compared with full-court and superiorities/inferiorities tasks. Notably, mid-court and ¾ court tasks were the categories with lower ACC_min−1_, DEC_min−1_, and PL_min−1_ values.

The physiological parameters were classified through cluster analysis into two groups: (1) “low physiological load” and (2) “high physiological load.” These clusters were strongly associated with the predictor variables HR_AVG,_ TDS_min−1_, and HSR_min−1_, with predictor importance values of 1.00, 0.96, and 0.31, respectively. The model quality represented by the average silhouette was 0.5, indicating a moderate model quality (Table 4).

**Table 4:** Cluster analysis identifying exercise groups based on physiological and biomechanical load variables.

Cluster	Variable	Importance of the cluster predictor	Low physiological load	High physiological load	
Physiological cluster	HR_AVG_ (bpm)	1.00	133.98 ± 20.04	161.45 ± 14.20	
	TD_min−1_ (m)	0.96	69.22 ± 21.55	104.35 ± 15.48	
	HSR_min−1_ (m)	0.31	5.50 ± 5.49	16.94 ± 13.26	
	Sample size (*N*)		757	1,222	
	Sample percentage (%)		38.3%	61.7%	
	Bayesian information criterion (BIC)		3,024.18		
	Average silhouette		0.5		
Cluster	Variable	Importance of the cluster predictor	Low biomechanical load	Medium biomechanical load	High biomechanical load
Biomechanical cluster	ACC_min−1_ (m)	1.00	3.30 ± 2.63	8.92 ± 3.61	19.72 ± 7.70
	DEC_min−1_ (m)	1.00	2.95 ± 2.24	7.76 ± 3.07	16.99 ± 5.91
	PL_min−1_ (a.u.)	1.00	1.27 ± 0.40	1.93 ± 0.31	2.34 ± 0.45
	MP (W/kg/min)	0.21	209.26 ± 65.24	304.24 ± 38.20	518.51 ± 709.78
	Sample size (*N*)		857	966	156
	Sample Percentage (%)		43.3%	48.8%	7.9%
	Bayesian information criterion (BIC)		2,849.59		
	Average silhouette		0.5		

In terms of biomechanical load, three distinct clusters were identified: (1) “low biomechanical load,” (2) “medium biomechanical load,” and 3) “high biomechanical load.” The clusters were strongly associated with the predictor variables: ACC_min−1_ (1.00), DEC_min−1_ (1.00), PL_min−1_ (1.00), and load–power met (0.21). The average silhouette score for this model was also 0.5, indicating a moderate quality (Table 4).

Table 5 presents the average physiological and biomechanical values by training task category, based on the cluster analysis. Superiorities/inferiorities, followed by full-court exercises, exhibited the highest physiological and biomechanical cluster averages, while introductory exercises showed the lowest. Finally, analytical, mid-court, and ¾ court exercises showed a middling physiological and biomechanical cluster average.

**Table 5:** Descriptive analysis of physiological and biomechanical load for each training task category.

Training task categories	Physiological load		Biomechanical load	
	Mean ± SD	Median	Mean ± SD	Median
Introductory technique-activation exercises (20 × 20, 28 × 20, 40 × 20)	1.10 ± 0.30	1	1.10 ± 0.37	1
Analytical situations	1.46 ± 0.50	1	1.66 ± 0.60	2
Exercises in mid-court (20 × 20)	1.53 ± 0.50	2	1.56 ± 0.66	1
Exercises in ¾ of the court (28 × 20)	1.67 ± 0.47	2	1.60 ± 0.55	2
Exercises in full court (40 × 20 m)	1.83 ± 0.38	2	1.81 ± 0.60	2
Superiorities/inferiorities (40 × 20)	1.92 ± 0.27	1	2.08 ± 0.62	2

**Figure 1:**
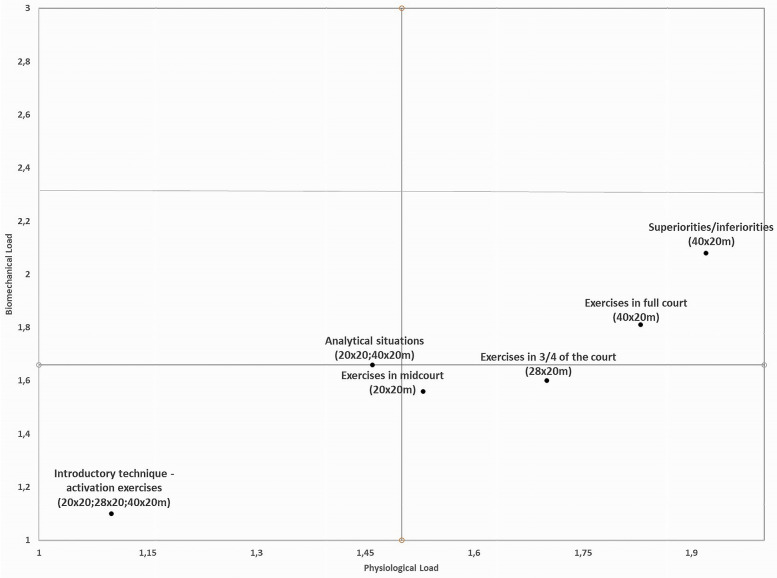
Visual representation of the physical demands for each exercise category in terms of physiological and biomechanical load.

Based on the average values reported in Table 5, each training task category was placed within a quadrant according to its level of effort. Accordingly, Figure 1 provides a visual representation of the physical demands of each exercise category in terms of physiological and biomechanical load. Introductory exercises clearly exhibited the lowest demands, characterized by both low physiological and biomechanical load. Analytical, mid-court, and ¾ court exercises were associated with moderate-to-high physiological demands and low biomechanical demands. Notably, superiority/inferiority and full-court exercises were the most demanding, showing high physiological and moderate biomechanical loads.

## Discussion

This study aimed to analyze the workload typology of professional futsal training tasks to better understand their impact on the physiological and biomechanical demands placed on players. Additionally, the study sought to categorize these training tasks using a visual quadrant-based approach to more clearly illustrate the types of efforts associated with each task category.

As expected, the results demonstrated that training task typologies can be profiled according to the training objectives, number of players involved, and field dimensions. In general, warm-up and introductory exercises imposed lower loads than other task categories. However, based on previous research in soccer, it was somewhat unexpected to observe that exercises conducted in 20 m × 20 m or 28 m × 20 m spaces produced lower physiological and biomechanical load values than those performed in larger spaces. This trend, also reported in a previous study (8), suggests that more than the number of players or the total area, the relative area per player is the primary variable determining the workload of each training task.

The introductory exercises showed lower physiological demands, primarily due to lower values for the HR_AVG_ variable. As expected, this suggests that these exercises are intended to prepare players for subsequent tasks beginning in a smooth and controlled manner. Meanwhile, it is important to note that HR_AVG_ values were very similar across the other task categories, which limits their ability to distinguish the specific physiological impact of each task (15). Therefore, rather than relying solely on HR_AVG_, it is advisable to combine different internal and external load variables to better capture the physiological impact of each training task. In line with previous findings in rink hockey (10), the combination of HR_AVG_, TD, and HSR appears to be more appropriate for classifying the physiological demands of futsal training tasks.

Regarding the variations in physiological demands between training tasks, results indicate significant differences across different categories of tasks, with the highest values TD_min−1_ and HSR_min−1_ values being observed in superiorities/inferiorities and full-court tasks. Interestingly, both categories are typically performed in larger spaces (40 m × 20 m), which aligns with recent findings (9) which found that larger areas and particularly larger relative areas per player tend to be associated with higher match demands. Similarly, a previous study (8) reported that full-court training tasks are the most demanding, largely due to the constant requirement for high-intensity actions and HSR enabled by the available space.

In line with the previous findings, the training tasks that use superiorities/inferiorities in full court promote the occurrence of high values of HSR_min−1_ and TD_min−1_. Beyond the defensive players need to cover more than one opponent and protect the goal, the available space allows players to cover great distances with the ball to pin the immediate defenders and create secure passing lines to the attackers free of opposition. Likewise, the space available in these tasks is higher than in the competitive context increases the relative area per player, thereby providing more opportunities to run with the ball, create passing and shooting lines (attackers), or recover positioning in relation to the ball (defenders) (8). Therefore, if the coaching staff aims to increase physiological efforts in a training session, the use of training tasks with superiority/inferiorities in medium to full courts seems to be a good option.

Regarding mechanical aspects, the results from the present study indicate that tasks involving superiorities/inferiorities also impose higher biomechanical loads (moderate-to-high) compared with other task categories. In line with previous studies in this sport, ACC and DEC metrics are particularly relevant during official futsal matches (16, 17). In this context, the results are related to the available space of play and the increased opportunities for players to cover longer distances at high intensity. The need to change direction, accelerate, and decelerate is crucial for actions such as breaking away from a defender or preparing to shoot (2).

In fact, the relative space per player used in these tasks was higher than in the competitive matches. Thus, the use of numerical superiority and inferiority training tasks in large spaces can be considered the most demanding and may help prepare players for the most demanding periods of the match (18). Similar findings have been reported in elite rink hockey regarding the influence of ACC and DEC metrics on manipulating biomechanical efforts (10). Additionally, coaches should prioritize these exercises as they replicate common offensive transitions in matches, which typically correspond to the most physically demanding moments (19).

In football studies, it has been shown that field size and the number of players significantly affect physiological and physical responses during small-sided games (20). Previous research has also shown that small-sided games in football, designed specifically to improve technical and tactical skills, can increase physiological and biomechanical demands (21). Similarly, our study found that the size of the playing space influences the physical demands of the task, as tasks performed in larger spaces (full-court and superiorities/inferiorities exercises) imposed greater physiological and biomechanical loads than exercises in smaller spaces. This trend was also observed in another study (7) which reported that small-sided games in the midcourt had lower impact values compared with ¾ court and full-court exercises.

However, unlike in football, small and medium spaces in futsal require fewer biomechanical actions than larger spaces. This may be attributed to the specific characteristics of the sport and the number of participants in the tasks, which are typically fewer than in football exercises. One prior study (9) demonstrated that, among youth futsal players, 3 × 3 tasks elicited higher biomechanical efforts than 2 × 2 tasks in a 20 m × 20 m pitch size. Therefore, constraining the relative space per player appears to be the most adequate strategy for understanding the physiological and biomechanical requirements of each task. This approach has also been suggested for rink hockey to adjust the biomechanical demands of training tasks according to those experienced in competition (10). This evidence highlights the need to understand the physical demands of exercises compared with match demands.

Future research should include a broader sample and carefully differentiate task variables to validate these findings. The main limitations of this study include the lack of information regarding the physiological and biomechanical demands of gameplay and the absence of positional categorization. Since playing positions may influence the load experienced during training and competition, this could introduce variability in interpreting the results (22). Comparing these findings with match data and considering positional roles may be crucial to better understanding how training translates to game demands.

Interestingly, the exercises with the longest practice time (introductory, analytical, and mid-court exercises) show the highest external load values particularly in terms of metabolic power. This finding aligns with a previous study (16), which indicated that metabolic power may be a key variable for characterizing the volume of effort during official matches.

Scientific knowledge aims to enhance field practices through evidence, while training load monitoring tools are essential for improving performance and reducing injury risk (23). Categorizing tasks that impose higher physiological and biomechanical demands enables coaches to adjust and structure training loads to improve players’ physical preparation. Analyzing training load by using a four-quadrant-based approach offers a clear and practical way to bridge the gap between sports scientists and coaches and, at the same time, between training and competition. In addition to characterizing exercise categories, this approach also allows for individualized player monitoring, providing coaching staff with simple and reliable information to support decision-making.

The identification of the physiological and biomechanical demands of different training tasks enables a precise understanding of training load typology in futsal players. By determining how each type of exercise impacts the athlete, the coaching staff can adjust training sessions within microcycle planning, organizing training loads according to the competitive phase and performance objectives. Furthermore, the visual quadrant approach used in this study enhances the understanding of physical demands and allows coaches to assess the impact of training tasks, aiming to replicate real match conditions and thereby promote an effective transfer of physical and tactical capacities to the competitive context.

## Conclusions

This study demonstrated that different categories of futsal training tasks impose distinct physiological and biomechanical loads on players. Superiority/inferiority and full-court exercises elicited the highest demands, underscoring the need to recognize their significant influence on player workload. These findings are consistent with previous research in both futsal and football, providing a strong basis for the optimal distribution of training loads in futsal. The four-quadrant-based approach proved to be a valuable tool for characterizing and classifying the physiological and biomechanical demands of the analyzed training task categories.

## Data Availability

The raw data supporting the conclusions of this article will be made available by the authors, without undue reservation.
